# Is There an Employment Advantage for Immigrant Women Who Marry Natives in Italy?

**DOI:** 10.1007/s10680-025-09734-w

**Published:** 2025-07-01

**Authors:** Adda Carla Justiniano Medina, Marie Valentova

**Affiliations:** Living Conditions Department, Luxembourg Institute of Socio-Economic Research, Luxembourg University, Esch-Sur-Alzette, Luxembourg

**Keywords:** Intermarriage premium, Selection bias, Instrumental variables, Truncated sample, Combined method, Immigrants’ integration

## Abstract

In this study, we assess the impact of intermarriage on employment outcomes among immigrant women, compared with endogamous (immigrant women married to immigrants). We measure employment outcomes using three variables: a binary outcome (employed or not), a continuous outcome (average weekly hours of work), and a proxy for underemployment. The linear probability model reveals that intermarried immigrant women are nearly 8 percentage points less likely to be employed than their endogamous counterparts. With regard to the intensity of employment, there are no significant differences for women in both marriage types in their probability of being underemployed or in the hours worked.

## Introduction

Over recent years, developed economies have been increasingly affected by the growing inflow and number of immigrants. In 2021, foreign-born people represented around 8.4 percent of the European Union population (EUROSTAT, 2022), impacting different aspects of the receiving country, such as the labor market (wages and employment), the supply and demand for goods and services and demographic trends, such as the fertility rate, and the marriage and divorce rates (Chiswick & Hatton, 2003; Furtado, 2016; Vignoli et al., 2017). A notable insight is that while marriage rates in the EU are declining, the intermarriages rates between foreign-born people and natives are increasing (Johnson & Kreider, 2013; Lanzieri, 2012).

As the proportion and diversity of the populations in European countries grow, so does concern regarding the integration of the foreign-born into the receiving societies. This is also evidenced by a survey, in which around 68 percent of surveyed EU citizens identified it as a priority (Eurobarometer, 2022). Upon arrival, foreign-born people are likely to have limited local-specific information and limited local networks, and may also have a limited grasp of the local language compared with natives (Chiswick, 1978). In most receiving countries, foreign-born individuals generally experience wage and employment penalties compared to natives (Vaaluavo & Rask, 2022 and 2024; Ingwersen & Thomsen, 2021; Choe & Van Kerm, 2018; Ballarino & Panichella, 2017). However, these gaps tend to diminish over time as foreign-born individuals integrate in the receiving country (Chiswick & Miller, 2002; Meng & Meurs, 2009). Intermarriage to a native is often seen as a mechanism that accelerates integration (see, among others, Meng & Meurs, 2009; Furtado & Theodoropoulos, 2009; Nottmeyer, 2010; Georgarakos & Tatsiramos, 2009).

Intermarriage is often considered both an indicator and facilitator of integration, reflecting mutual acceptance and willingness to integrate with each other (Alba & Golden, 1986; Kalmijn, 1998; Muttarak, 2004). Furthermore, it can accelerate the integration process of the immigrant partner within the host country by improving employment outcomes through access to the native partner’s local-specific knowledge and social networks (Dribe & Lundh, 2008; Furtado & Theodoropoulos, 2009; Gevrek, 2009; Kantarevic, 2004; Nottmeyer, 2010).

The existing literature identifies two primary channels through which intermarriage enhances labor market integration: first, the information that natives share with their immigrant partners, for example, about local culture, institutions, idiosyncrasies and language (e.g., Meng & Meurs, 2009), and second, expanded access to the social networks of the native partner (e.g., Furtado & Theodoropoulos, 2010).

The vast majority of the literature on intermarriage and labor market focus on wages, specifically for immigrant men, often reporting a wage premium.^1^ However, it is inconclusive whether this derives from positive selection into intermarriage (Dribe & Nystedt, 2015; Kantarevic, 2004; Nottmeyer, 2010) or a positive effect of intermarriage (Gevrek, 2009; Meng & Gregory, 2005; Meng & Meurs, 2009). Given the distinct factors that shape immigrant women labor market, these findings might not be directly extrapolated to them (Livingston, 2006).

Research on intermarriage’s impact on immigrant women’s labor market outcomes remains limited and inconclusive. While studies from the USA and Sweden suggests that intermarriage may lead to penalties in both wages and hours worked for immigrant women (Basu, 2015; Bevelander & Irastorza, 2014), they also found an intermarriage premium in terms of employment. In contrast, studies from Australia and France observed an intermarriage premium in wages (Meng & Gregory, 2005; Meng & Meurs, 2009).

Existing research on intermarriage and labor market outcomes for immigrant women focuses on few countries, highlighting a significant research gap on newer migration destinations in Southern Europe, particularly Italy.^2^ Unique migration patterns, illegal migration, the feminization of immigration, growing labor market participation of immigrant women and rising intermarriage rates in these regions highlight the relevance for study (Ballarino & Panichella, 2018). By 2008, nearly 15 percent of marriages in Italy involved an immigrant woman, with this figure rising by an average of 2 percent per year between 2000 and 2011, while natives’ marriages declined by nearly 3 percent (ISTAT, 2020, 2022). However, most academic attention to intermarriage in Italy has focused on its causes rather than its effects (Adda et al., 2020; Azzolini & Guetto, 2017; Guetto & Azzolini 2015; Serret & Vitali, 2014; Vignoli et al., 2017).

In view of this context, the aim of this article is to address the under-researched aspects of the relationship between intermarriage and the labor market of immigrant women, focusing on whether intermarried immigrant women in Italy are more or less likely to be employed, work longer hours or experience underemployment compared to their endogamous counterparts. To achieve this, it considers women’s self-declared employment status (being employed or not), the intensity of employment (hours worked) and the likelihood of underemployment (a person working 30 h a week or less, while working more hours would be preferred but cannot find a full-time job or work more hours in their current employment).

This paper contributes to the literature on the association between intermarriage and labor market outcomes in several ways. It is the first study to examine three different employment outcomes—employment, employment intensity and underemployment—simultaneously. This approach provides a more nuanced understanding of the complex relationship between intermarriage and the labor market integration of immigrant women, shedding light on the under-researched phenomenon of involuntary lower labor market engagement among immigrant women.

A key contribution of this study is its exploration of the mechanisms and heterogeneity underlying this relationship. Specifically, it incorporates interaction terms for various factors, including place of origin (EU, Eastern Europe and non-EU), regional unemployment rates, wife’s education, age gap within the marriage, migration reason and the presence of dependent children. This allows for a deeper analysis of how intermarriage affects different groups of immigrant women.

Another contribution is that by using a unique dataset that surveyed immigrants in Italy during a period of financial crisis, this paper also examines how the economic downturn differentially impacted immigrant women based on their type of marriage. Additionally, it highlights how regional unemployment rates during the 2007 financial crisis may have influenced immigrant women’s labor market outcomes differently depending on their marital status and how gender role ideologies in their origin countries, relative to the host country, shape these outcomes. While the analysis is based on 2008 data—offering important historical insights—it acknowledges the limitations of applying these findings to evolving labor market contexts.

By focusing on Italy, the study broadens the geographical scope of research on intermarriage and employment outcomes, which contributes to the existing knowledge. Italy’s distinctive demographic and social context provides valuable insights into how intermarriage influences the labor market integration of immigrant women in Southern Europe.

## Theories and Existing Evidence

### Theoretical Background

There are different theories and arguments that could help explaining the relationship between intermarriage and the various employment outcomes. The productivity theory and the social theory converge in suggesting a positive role of intermarriage in facilitating integration and access to native resources.

The productivity theory focuses on the acquisition of local knowledge and human capital (Dribe & Lundh, 2008; Gevrek, 2009; Nekby, 2010; Nottmeyer, 2010), while the social theory emphasizes the role of networks and local labor market awareness (Aguilera, 2002; Furtado & Theodoropoulos, 2010). Additionally, natives’ recommendations may further enhance hiring prospects. Intermarriage may signal also greater attachment and adaptability to the receiving country’s labor market ultimately enhancing labor market prospects (Furtado & Theodoropoulos, 2010).

Conversely, according to the selection theory, intermarried immigrants represent a self-selected subset of married immigrants possessing labor market skills highly valued in the native marriage market, such as language proficiency, communication, knowledge of local customs and physical appearance (Kantarevic, 2004). This theory contrasts the social and productivity theories due to potential reverse causality, where better integration may lead to intermarriage, rather than the other way around. Thus, any positive effects may be due to unobserved characteristics rather than intermarriage itself.

Alternative perspectives suggest that intermarriage can negatively impact immigrant women’s labor market outcomes due to gender-based division of labor (Becker, 1991). Families’ utility maximization assigns domestic roles to the lower-earning spouse, usually the wife, particularly when husbands have labor market advantages. If native husbands possess labor market advantages compared with wives—which may be particularly the case in intermarriage—this could negatively affect immigrant women’s participation in the labor market, the hours they work, and it could increase their likelihood of underemployment.

There are various reasons that may lead to underemployment: the first one being traditional gender roles, which often assign women primary responsibility for caregiving, leading them to seek part-time or flexible jobs that accommodate their domestic duties (Cha, 2010; Misra et al., 2011) and secondly, gendered occupational segregation, where women are often concentrated in industries or occupations that offer fewer hours, lower pay and precarious employment, such as retail, hospitality or caregiving (England & Folbre, 2003). Gender and motherhood discrimination, where assumptions that women or mothers prefer part-time work, might limit women’s working hours or job quality (Correll et al., 2007). However, economic constrains and job availability might impact underemployment, where flexible labor demand disproportionally affects women (Kalleberg, 2000).

Along similar lines, the bargaining power theory asserts that the bargaining power of each spouse intertwines with the gender-based division of labor, as elucidated by Naldini and Solera (2018) and Lundberg and Pollak (1996). In an intermarriage, the native partner could have greater bargaining power, stemming from higher income, and this could accentuate the gender-based division of labor (Basu, 2017). Moreover, as the bargaining power may depend on the alternatives spouses have outside of marriage, immigrant women could be further disfavored if intermarriage is the only alternative to obtain legal status (Nottmeyer, 2011).

Negative selection may also play a role. Several studies—including those by Scoppa and Stranges (2019), Pessin and Arpino (2017), Blau (2015), Crompton and Lyonette (2005), Kangas and Rostgaard (2007), and Haller and Hoellinger (1994)—suggest that gender role norms in the country of origin can influence immigrant women’s participation in the labor market of the host country. Additionally, native husbands and immigrant wives may match based on their gender role preferences (Basu, 2015). Native men with traditional gender role views may prefer immigrant women from societies with similar norms (Glowsky, 2007). Conversely, the prospect of a more equal division of gender roles may encourage immigrant women to marry native partners (Rodríguez-García et al., 2015).

The family investment theory (FIT) suggests that endogamous immigrant women initially enter the workforce to support their husband’s integration into the host country’s labor market. As an immigrant husband accumulates local human capital, his labor force participation tends to increase, while that of their wife tends to decrease (Baker & Benjamin, 1997; Blau et al., 2003; Worswick, 1999). Consequently, this would reflect a negative impact on the labor market involvement of intermarried women due to reduced financial constraints.

Intermarriage can be influenced by a variety of factors, such as economic status, education, cultural integration, the local marriage market and expected division of gender roles in a couple, with notable gender differences for immigrant men and women.

Economic status is a significant factor for both genders but operates differently. For immigrant men from lower socioeconomic backgrounds, intermarriage is often seen as a strategy to achieve social mobility, (Furtado & Theodoropoulos, 2011). Similarly, immigrant women with fewer economic resources may see intermarriage as a pathway to financial security (Chiswick & Houseworth, 2011). Conversely, immigrant women with economic independence are less likely to intermarry, as they do not view marriage as a financial necessity.

The status exchange theory suggests that immigrant women might trade their higher education for the social status of marrying a native partner, who may be less educated. This exchange could provide better integration opportunities and economic benefits in the host country and social mobility and secure a stable economic future (Chiswick & Houseworth, 2008).

The marriage market influences intermarriage rates, especially where there are fewer partners from one’s own ethnic group (González-Ferrer et al., 2018). Assortative mating plays a significant role in intermarriage patterns as highly educated women are more likely to have intermarry with similarly educated natives (Kalmijn, 2012; Qian & Lichter, 2007).

Immigrant men and women who are more integrated into the host society's culture, language and values are more likely to intermarry (Alba & Nee, 2003; Kalmijn, 1998). Conversely, strong ethnic identity may serve as a barrier to intermarriage, particularly for men who strongly adhere to their cultural traditions. Migrant women often exhibit more flexibility in blending their ethnic identity with that of the host society, leading to higher intermarriage rates (Min & Kim,^ 2009^).

However, Elwert (2020) highlights the significance of other elements when choosing a co-ethic or native partner such as education, income, age and prior relationships. Natives with lower status in this market may be more inclined to marry other lower-status individuals, including immigrants.

### Empirical Evidence for an Employment Intermarriage Premium. A Focus on Immigrant Women^3^

Dribe and Lundh (2008) were among the first to include women in their analysis, finding a strong positive association between intermarriage, earnings and employment for immigrant women in Sweden. However, they did not address endogeneity, leading to sub-selection of intermarried immigrants. Also in Sweden, Bevelander and Irastorza (2014) found an employment premium for intermarried women, controlling for pre- and post-marriage employment to account for endogeneity.

Vaalavuo and Rask (2022, 2024) analyze the labor market outcomes of male and female immigrants in Finland. Focusing on earnings and employment trajectories, the authors found that among male immigrants, earnings disparities by country of origin were smaller when analyzed separately by partnership status (no partner, native partner or immigrant partner). Among immigrant women, group differences remained stable over time, with Somalian and Middle Eastern women being notable outliers to the positive earnings trend. Only when partnered with a native-born Finn did these groups converge to the trend observed among other immigrant women. The authors conclude that they could not fully support the hypothesis that native partners generally provide immigrants with social capital beneficial for the labor market. They also acknowledge that their analysis does not account for selection into partnerships or mixed partnerships. Additionally, the study measures partnership status at the time of immigration, excluding observations where the partnership was formed post-immigration.

Basu (2015) examined intermarriage premiums on earnings, working hours and employment among immigrant women in the USA. While employment estimates showed a positive intermarriage premium, earnings and working hours exhibited a penalty. The author accounted for endogeneity via instrumental variables. Studies on working hours of immigrant women and the FIT include Baker and Benjamin (1997) in Canada and Blau et al. (2003) in the USA. Both found that on arrival, the labor supply hours among endogamous immigrant women are greater than those of their intermarried counterparts.

Ballarino and Panichella (2018) compared various employment outcomes for immigrant women, focusing mainly on their families and their migration strategy (migrated alone, first mover, tied mover, joint mover or intermarried). They found that intermarried immigrant women experienced the highest employment penalty in Italy and Spain; in fact, higher than for all other categories of immigrant women.^4^

Nottmeyer (2011) examined bargaining power and labor supply in intermarried immigrant households in Germany, finding that intermarriage rebalanced bargaining power in favor of women. In contrast, Basu (2017) suggested that intermarriage in the USA could reinforce gender specialization, disadvantaging immigrant women. Notmeyer also compared intermarried and cohabiting couples, considering both husband and wife employment outcomes, while Basu addressed endogeneity via instrumental variables estimation.

The articles that explore the intermarriage premium in labor market outcomes typically apply two approaches: One is grounded in the productivity theory usually employing ordinary least squares (OLS) estimation and the other on self-selection theory, using fixed effects methods, for longitudinal data or two-stage least squares (2SLS) for cross-sectional data.

## Italian Context

Italy began experiencing notable immigration relatively recently, compared with other countries that have a tradition of receiving migrants. A series of events have characterized the increasing inflow of immigrants in Italy by decades. Briefly, the first waves of immigrants arrived after the international crisis in the 1970s, a period during which traditional receiving countries began imposing restrictions on immigration. Italy became an option for immigrants who could no longer migrate to these other countries.

Inflows of immigrants became more significant in the 1980s, with arrivals from Iran and former Yugoslavia reaching as many as 100,000 per year (Reyneri, 2001). The 1990s saw further inflows due to the Balkan war (Del Boca & Venturini, 2005). Additionally, the enlargement of the European Union in the 2000s contributed to increased immigration from Eastern European nations (Fusaro & Lopez-Bazo, 2018).

It is essential to acknowledge that Italy faces a notable challenge with unregulated immigration, as highlighted by scholars such as Triandafyllidou (2010), Reyneri (2001), and Mingione and Quassoli (2000). Italy notably has some of the strictest regularization and citizenship regulations in the European Union.^5^ The majority of immigrant women in Italy find employment in low-skilled roles within the domestic service sector, as discussed by researchers such as Campani (2007), Bettio et al. (2006), Venturini and Villosio (2006), and Andall (1992). Yet this is a sector particularly susceptible to unregulated labor practices. Regularization through work has occurred via amnesties, which have been established sporadically and irregularly over the years. Hence, for many undocumented immigrants in the country, marriage often emerges as the sole viable avenue toward achieving regularization and citizenship status (Adda et al., 2020; Azzolini & Guetto, 2017).

According to the national census, in 2011 there were 4,027,627 immigrants in Italy; approximately 53 percent of them were women. Recent statistics for 2023 show that the overall ranking of immigrant women remains largely similar relative to the year 2008, although there have been some shifts in the composition of the largest groups.^6^ The most numerous groups of women in 2023 are Romanians (a total of 616,204, representing the 67% of all Romanians), Albanians (203,782, 49% women), Ukrainians (192,350, 77% women), Moroccans (190,572, 46% women), Chinese (152,045, 49.5% women), Filipinos (90,346, 57% women) and Moldovans (73,026, 66.5% women). However, significant changes in the rankings hav eoccured, with women from India, Poland, Nigeria, Sri Lanka, Egypt, Bangladesh and Pakistan increasingly replacing Latin American (Peruvian, Brazilian, Ecuadorian) and Balkan (Serbian, Montenegrin and Kosovar) women in the top spots.

For example, in 2023, the numbers included Indian women (70,592, 42% of Indians), Polish women (55,631, 75% of Poles), Nigerian women (53,030, 43% of Nigerians), Sri Lankan women (52,223, 47% of Sri Lankans), Egyptian women (49,865, 28.6% of Egyptians), Bangladeshi women (49,783, 27.3% of Bangladeshis) and Pakistani women (39,375, 27.3% of Pakistanis). Meanwhile, the number of Tunisian women (38,196, 37.3% of Tunisians) has seen relatively smaller increases. In contrast, Brazilian women (35,323, 69.1% of Brazilians), Ecuadorian women (35,208, 55.7% of Ecuadorians) and Serbian/Montenegrin/Kosovar women (33,191, 47.9% of these nationalities) have experienced a decrease. Notably, migration from Eastern European countries is predominantly female, with women comprising nearly 70% of total migrants. In contrast, migration from Asian and North African countries tends to be male-dominated, possibly reflecting the more traditional gender roles prevalent in those societies.

The occupations of immigrants in Italy differ between regions, due to the local structure of the economy. Domestic labor is relevant in the central cities of Bologna, Florence, Rome and Milan and in the largest cities of the southern regions (Allasino et al., 2004).

In the realm of attitudes toward women’s employment, it becomes evident that when examining Italy, a relatively small proportion of the population supports the idea of equal participation of both men and women in the labor market compared with other European countries (Arpino et al., 2025). This conclusion is supported by the empirical analyses of Colonna and Marcassa (2015), in which they found that the participation of married women in the labor market in Italy is the lowest among EU countries.

When an immigrant woman is married to an Italian native, she has a favored path to citizenship, and can apply after 2 years of residence in Italy (1 year if there are children). However, this is not the case for endogamous women, where their permit of stay may depend on their husband’s status. After 5 years of residence, if they fulfill other requirements (income, language and conviction-free background), they can apply for a permanent permit of stay or for citizenship after 10 years.

## Hypotheses

Intermarriage could aid the acquisition of local-specific human capital and provide access to native networks increasing awareness of job openings. However, a native husband often holds a comparative advantage in the labor market due to his proficiency in the local language, familiarity with the local labor market and established social capital. This can reinforce traditional gender roles, where wife focuses on domestic duties while the husband engages in market work.

Furthermore, the husband’s advantage can result in higher bargaining power within the marriage, especially if the wife’s legal status depends on her husband, pressing her to comply with the husband preferences. Additionally, the reduced financial pressures in an intermarriage may diminish the immigrant wife’s urgency to participate in the labor market, unlike her endogamous counterparts, who may experience greater economic necessity. Therefore, it is hypothesized that intermarriage is negatively related to employment for immigrant women compared to their endogamous counterparts (H1).

Regarding intensity of labor market participation, measured by the number of hours worked, endogamous women may work longer hours than their intermarried counterparts, due to greater financial need. This economic necessity likely leads to increased working hours, while intermarried women might experience more gender specialization. Therefore, the hypothesis posits a negative relationship between intermarriage and labor working hours (H2).

Finally, the elevated status of native husbands may translate into higher bargaining power, which could limit their wives’ involvement in labor market work and result in more hours dedicated to domestic tasks. This dynamic can result in greater underemployment. Therefore, we hypothesize that intermarriage is associated with higher underemployment for intermarried women compared to their endogamous counterparts (H3).

## Data and Descriptive Statistics

The data used for the analysis are sourced from the Italian national statistics office, *Istituto Nazionale di Statistica* (ISTAT). We utilized the survey “Life Conditions of Foreign Families in Italy” (*Condizioni delle famiglie con stranieri*), a unique survey specifically dedicated to understanding the living situation of the foreign population residing in the country. The survey was conducted in the eight main languages spoken among the foreign community, in addition to Italian and English. It was carried out in 2008 and 2009.

In our analyses, we focus on a subsample of first-generation immigrant women aged 18 to 65 who migrated as adults (after age 18) and have an identifiable country of birth. It should be noted that women who migrate as adults and do not go through the Italian school system (the selected sample) tend to have lower levels of integration and poorer proficiency in the host language, which negatively affects their labor market outcomes. However, we restricted our sample to women who migrated after the age of 18, for several reasons. First, the number of women who migrated as children (before age 18) is relatively small, comprising only 10% of the full sample (290 out of 2,834 women), and the number of those who intermarried is considerably smaller (29 intermarried women, 10%). Including them in the analysis would not allow for statistically robust results. Moreover, free school education in Italy typically lasts until age 18. Therefore, as mentioned earlier, women who migrated as children are likely to have been exposed to educational structures and systems that make them fundamentally different from those who migrated as adults. Moreover, child migrants are also significantly younger in the sample (average age 26, compared to 36 for adult migrants) and have spent more years in the country, as reflected by the years since migration (YSM) variable, which might hint that they are not representative of the recent migrant flows driving the feminization of migration in Italy, which is mainly composed by adults.

Additionally, women who migrated as children typically did so due to parental decisions; therefore, even if they move for family reasons, it differs from adult migrants who chose to move for family reasons related to marriage. Including both groups would introduce conceptual complexity in interpreting migration motives.

To ensure our selection does not bias the results, we provide a sample balance table (please see Appendix, Table 10) comparing women who migrated as children to those who migrated as adults. The sample balance table indicates many major differences between the groups. However, we also re-estimated the analysis including the 290 child migrants (please see Appendix, Table 11). The results remained consistent, likely due to the small size of the child migrant subsample. Including this subgroup would introduce complexity without significantly increasing the statistical power needed for a detailed analysis.

Therefore, we focus on women who migrated after the age of 18, who are a more homogeneous and relevant sample for examining the concerns related to immigrant women within the context of the feminization of immigration in Italy and the concerns addressed in this article.

Based on these arguments, the sample used in this paper comprises 2544 observations, while the sample of those employed consists of 1020 observations. We also used register data from ISTAT for 2008 to create the instruments for group size, sex ratio and percentage of foreign men. Lastly, we used the dataset from the fifth wave of the World Values Survey (WVS 2005–2009) to create the proxy for attitudes toward women’s employment. A detailed description of the variables and instruments can be found in the “variables description” subsection in the “Econometric Framework” section.

Table 1 presents the descriptive statistics for immigrant married women. The left-hand columns show the full sample and the right-hand columns the sample of those who are employed, by type of marriage (intermarried and endogamous). Intermarried women have slightly lower levels of employment compared with their endogamous counterparts (37.5 versus 41 percent). Intermarried women on average have higher levels of education, are nearly two years older than women in an endogamous union and have the highest proportion of those who had spent more than 15 years in the country. Most intermarried women are from East European and non-European countries. A larger age gap (greater than 6 years) is more common among intermarried women (52%) compared to endogamous women (27%). The primary migration reason for intermarried women is work-related (49%), closely followed by family-related reasons (46%). As intermarried women possess more human capital and have spent more years in the country, this could suggest positive selection into intermarriage.

**Table 1:** Descriptive statistics of first-generation immigrant women in Italy (ages 18–65), by employment status and partnership type. Source: Istituto Nazionale di Statistica. Condizioni di Vita delle Famiglie con Stranieri (ISTAT) 2008–2009. https://www.istat.it/it/archivio/52405

	Full sample						Employed sample					
	Total (N = 2544)		Endogamous (N = 1850)		Intermarried (N = 694)		Total (N = 1055)		Endogamous (N = 781)		Intermarried (N = 274)	
Variable	Mean	S.D	Mean	S.D	Mean	S.D	Mean	S.D	Mean	S.D	Mean	S.D
Employment	0.40	0.49	0.41	0.49	0.37	0.48	–	–	–	–	–	–
Hours worked	–	–	–	–	–	–	33.55	11.93	33.53	12.32	33.62	10.75
Underemployed	–	–	–	–	–	–	0.17	0.38	0.18	0.38	0.14	0.35
*Education level*												
Low secondary	0.45	0.50	0.53	0.50	0.26	0.44	0.38	0.48	0.43	0.50	0.22	0.41
Upper secondary	0.43	0.49	0.38	0.49	0.55	0.50	0.48	0.50	0.45	0.50	0.55	0.50
University	0.12	0.32	0.09	0.29	0.19	0.40	0.15	0.35	0.11	0.32	0.24	0.43
Average age	37.15	8.89	36.71	8.76	38.32	9.12	37.63	8.10	37.54	8.07	37.90	8.20
Residing in north	0.45	0.50	0.49	0.50	0.37	0.48	0.47	0.50	0.48	0.50	0.45	0.50
NDC	0.47	0.66	0.49	0.68	0.44	0.63	0.35	0.61	0.36	0.62	0.31	0.57
*YSM categories*												
0–5	0.37	0.48	0.37	0.48	0.38	0.49	0.35	0.48	0.37	0.48	0.30	0.46
6–10	0.37	0.48	0.37	0.48	0.35	0.48	0.38	0.49	0.38	0.49	0.40	0.49
11–15	0.15	0.35	0.15	0.36	0.13	0.34	0.14	0.35	0.14	0.35	0.15	0.36
> 15	0.11	0.31	0.10	0.30	0.14	0.34	0.12	0.33	0.12	0.32	0.15	0.36
*Place of origin*												
Western Europe	0.10	0.29	0.04	0.20	0.23	0.42	0.11	0.31	0.05	0.21	0.27	0.45
Non-EU	0.45	0.50	0.50	0.50	0.31	0.46	0.40	0.49	0.46	0.50	0.25	0.43
Eastern Europe	0.45	0.50	0.45	0.50	0.46	0.50	0.49	0.50	0.50	0.50	0.48	0.50
*Regional unemployment*												
Northeast (3%)	0.24	0.42	0.25	0.44	0.19	0.39	0.25	0.44	0.25	0.44	0.26	0.44
Northwest (4%)	0.22	0.41	0.23	0.42	0.18	0.38	0.22	0.41	0.23	0.42	0.19	0.39
Center (6%)	0.20	0.40	0.21	0.41	0.17	0.38	0.21	0.41	0.22	0.41	0.20	0.40
South (12%)	0.18	0.38	0.17	0.37	0.20	0.40	0.15	0.36	0.15	0.36	0.16	0.37
Islands (14%)	0.17	0.38	0.14	0.34	0.26	0.44	0.16	0.37	0.15	0.36	0.19	0.40
Age gap (> 6 years)	0.34	0.47	0.27	0.44	0.52	0.50	0.26	0.44	0.19	0.39	0.45	0.50
*Migrating reason*												
Family related	0.53	0.50	0.55	0.50	0.46	0.50	0.33	0.47	0.30	0.46	0.40	0.49
Work related	0.44	0.50	0.42	0.49	0.49	0.50	0.64	0.48	0.66	0.47	0.57	0.50
Other	0.03	0.18	0.03	0.16	0.05	0.22	0.03	0.18	0.03	0.18	0.04	0.19
Dependent children dummy	0.38	0.49	0.39	0.49	0.37	0.48	0.29	0.45	0.30	0.46	0.26	0.44
*Instruments*												
Pct. foreign men	0.09	0.04	0.09	0.04	0.08	0.04	0.09	0.04	0.10	0.04	0.09	0.04

The columns on the right-hand side of Table 1 present the descriptive statistics for the sample that is the focus of the underemployment exercise. Only those who reported a positive number of weekly hours of work (thus, only employed women) are included.

Underemployment rates are slightly higher for intermarried women Intermarried women in northern Italy tend to have higher levels of university education compared to their endogamous counterparts, with 24% holding a degree versus 11%. Both groups are similarly aged, averaging around 37 years and predominantly reside in northern Italy (48% for endogamous and 45% for intermarried women). Intermarried women also have slightly longer residency durations, with 40% having lived in the country for 6–10 years and 30% for over 10 years, compared to 38% and 26% for endogamous women, respectively. The majority of endogamous women come from Eastern Europe (50%) and non-EU countries (46%), whereas intermarried women are more likely to come from Western Europe (27%). Although work is the primary reason for migration for both groups, a higher percentage of endogamous women (66%) state to have moved for this reason compared to intermarried women (57%). In line with the second robustness check, we integrated specific husband-related attributes, including their logarithmic annual income and a proxy for attitudes toward women’s employment held by both husbands and wives.

## Econometric Framework

In order to estimate the association between intermarriage and employment, we use a Mincer equation of earnings, as the variables affecting earnings—education age and age squared as a proxy of experience—could also affect other employment outcomes (Mincer, 1974). Additional variables that could affect employment outcomes were also included.

$${\text{Employment\_outcome}}_{i} = \beta_{0 } + \beta_{1 } {\text{Married}} + X_{2 } \beta_{2} + \varepsilon_{i}$$

In the first part of the analysis concerning binary outcomes, we use a linear probability model (LPM) estimation as a baseline, since it provides the best linear approximation. In this way, we can establish the “raw” intermarriage premium or penalty for employment, meaning that intermarriage is treated as exogenous.

A common challenge in estimating the effects of marriage on employment concerns the potential issue of endogeneity. This arises because the type of union (endogamous or intermarriage) may also be correlated with unobserved variables (Kantarevic, 2004; Meng & Gregory, 2005). Furthermore, reverse causality between employment and the type of union is possible, in that being employed or unemployed may also affect the likelihood of meeting potential partners. For these reasons, there is a strong possibility that intermarriage is endogenous, either due to unobservable variables or reverse causality. To address potential endogeneity in intermarriage, we conducted a robustness check by using an instrumental variable approach with a measure that accounts for the marriage market in the respective region of residence as an alternative to the linear probability model (LPM). The instrumental variable (2SLS) approach addresses endogeneity by using an external instrument to isolate the variation in the endogenous variable that is uncorrelated with the error term, providing more accurate and unbiased estimates of causal effects (Wooldridge & Weeks, 2002). However, due to possible limitations of this instrument, the primary focus remains on the LPM analysis.

Additionally, we performed a second robustness check that augmented the estimated models by variables that could potentially affect the employment outcomes such as the partner’s income, homeownership and synthetic proxies for attitudes toward women’s employment. The results of these checks are discussed in the “Results” section, with further details provided in Appendix.

Furthermore, we conducted several heterogeneity analyses to explore how intermarriage interacts with factors such as place of origin, regional unemployment rates, the wife's education, the age gap within the marriage, reasons for migration and the presence of dependent children.

In the second part of the analysis, we examine the impact of intermarriage on employment intensity, specifically focusing on the weekly number of hours worked. This outcome variable poses a methodological challenge due to the “corner solution” problem, where a substantial number of observations have zero working hours because of not being employed. To address this, we use the double hurdle estimation method, which effectively handles the accumulation of zero values typical for individuals with no reported working hours (Wooldridge & Weeks, 2002). In the first step, the hurdle model, estimates the probability of having non-zero working hours. In the second step, it examines the intensity of employment—hours worked—among those who are employed.

Differently from the Heckman sample correction method, where the data are observed only for part of the sample (sample selection bias), in this case the working hours are presumably observed for all women, hence, the hurdle method seems appropriate.

The third part of the analysis consists of estimating the effect of intermarriage on underemployment. Since the underemployment variable is a binary, we use the LPM and Probit estimation method as an additional robustness check.

### Dependent Variables

We start by studying the effect of intermarriage on the extensive margin of employment; hence the first outcome is a binary variable that assess whether a women is employed. The employment status is self-assessed and equals 1 for a woman who stated they were working full-time, part-time or were self-employed full-time or part-time. In all other cases (e.g., unemployed, student, disabled, homemaker, inactive or pensioner), the variable equals 0. The information about employment was cross-validated by looking at the number of hours worked. About 98 percent of those who reported not being employed also reported zero working hours, which confirms the non-employed status.

The second outcome concerns the intensive margin of employment and is measured by the reported average hours of work in a week.

The third outcome is a proxy for underemployment, and we measure this by using a question on the reason for working 30 h or less per week. The variable is binary and equals 1 for a woman who works 30 h a week or less, who would prefer to work more hours, but cannot. The value of 0 is assigned for a woman who either works 30 h or less out of her own choice, or if she works more than 30 h a week.

### Independent Variables

The key independent variable measures intermarriage. It is categorical and equals 0 if an immigrant woman is married to another immigrant (endogamous), it equals 1 if a woman is married to a native (intermarriage). We included cohabiting partners and married partners in the same category, because they are defined in this way in the original dataset, and after cross-validating with the marital status, we find that less than 3 percent of the women in our sample were in a cohabiting union without being married. This proportion is too small to create a separate category.

In addition, the vector of variables X contains controls, such as age, and its squared term, as a proxy for experience. We divided age squared by one thousand, for illustration purposes. In order to retain enough observations and ensure harmonization between the education levels from the different countries, education is divided into three categories: up to lower secondary education, up to upper secondary education and university education.

Moreover, we include controls for other factors that could affect employment, such as the number of dependent children and the region of residence. Dependent children are defined as those under 6 years old, as in Italy, children of that age do not attend compulsory schooling. Therefore, they require a parent’s time, potentially reducing time to devote to employment (Lalive & Zweimüller, 2009; EC, 2008; Kleven et al., 2019). The region of residence is controlled via a binary variable, equal to 1 form women living in the northern regions, which are characterized by lower unemployment rates, and 0 for any other region.

Additionally, we also include a set of variables that could affect immigrants’ employment integration: years since migration and place of origin. An immigrant who has spent more time in the host country is typically better integrated. However, this relationship may not be linear, so we chose to categorize the time spent into ranges: 0–5 years, 6–10 years, 11–15 years and over 15 years. The country of origin was aggregated, in order to have enough observations in each category; while countries are diverse overall, those within the same category tend to share more similarities with each other. People from EU counties are generally allowed to live and work in Italy, whereas most individuals from Eastern European and non-EU countries are not (except for Poland and Romania). Moreover, Eastern European countries might share similarities due to their shared history, compared with non-EU countries. Non-EU countries are highly heterogeneous, with immigrants from these regions having diverse origins and relatively small numbers. As a result, breaking them down into further categories would likely reduce the sample size to an impractical level. Additionally, the original dataset aggregates individuals from Eastern European countries—such as Croatia and Serbia—into the non-EU category to comply with data protection rules due to their low incidence.

In the robustness checks, we incorporated partner characteristics, including salary, a home ownership indicator and a synthetic proxy for attitudes toward women’s employment for both partners. This synthetic proxy was created using an imputation method, where missing values were estimated based on the average of individuals with similar characteristics (Seiler & Heumann, 2013). We used the WVS (World Values Survey) question: “when jobs are scarce, men should have more right to a job than women” (strongly agree, agree, neutral, disagree, strongly disagree).

Additionally, the heterogeneity analyses include variables such as regional unemployment rate, an indicator of age gap within the marriage (larger than 6 years) and the reasons for migrating.

### Instrumental Variables

Estimating the impact of intermarriage on labor market outcomes requires addressing potential endogeneity concerns. Unobserved factors, such as language proficiency, may influence both marital choices and employment prospects. For instance, women with higher language skills might be more likely to interact with natives, increasing their chances of intermarriage and enhancing their employment opportunities. Additionally, reverse causality could exist; employment may provide greater opportunities to meet native men, thereby influencing marriage decisions.

To address this endogeneity, we utilize the percentage of foreign men in each region as an instrumental variable. This metric reflects the local marriage market dynamics, where a higher proportion of foreign men relative to the total male population increases the likelihood of endogamous unions and decreases the probability of intermarriage. This approach aligns with Furtado and Theodoropoulos (2009), who employed a similar instrument in their study of male immigrants. Furthermore, a higher concentration of foreign men may reinforce social norms favoring endogamy, as discussed by Kalmijn (1998).

Therefore, we expect this variable to positively influence endogamous unions and negatively affect intermarriage rates. Other variables to account for endogeneity of intermarriage in the literature are the group size and the sex ratio (Meng & Gregory, 2005; Meng & Meurs, 2009). In our case, the percentage of foreign men was proved to be stronger for our sample (comparatively higher F-statistic); therefore, we dismissed the other two instruments. More details about the conditions of the instruments are found in the robustness check.

## Results

### Intermarriage and Employment Likelihood

In the first step of our analyses, we performed an LPM estimation, the results of which are presented in Table 2, where the first model shows the raw intermarriage penalty for employment. It is negative and statistically significant at the 10% level of significance (−4.0 percentage points). Moreover, in the second model when the education and experience variables are added, the magnitude of the coefficient increases and it becomes highly significant (−8.0 percentage points). In the third model, in which we add the dummy variable for living in the north and the number of dependent children, the coefficient for intermarriage decreases slightly while it is still highly significant (to −7.0 percentage points). Lastly, in the fourth model, where we add the immigrant-specific controls—years since migration and place of origin—the coefficient increases again and it is highly significant (−8.0 percentage points). This finding supports our first hypotheses (H1) that intermarriage is related to a penalty regarding employment, compared to endogamous women.

**Table 2:** Linear probability model (LPM) estimates of employment among immigrant women in a partnership in Italy. Source: Istituto Nazionale di Statistica. Condizioni di Vita delle Famiglie con Stranieri (ISTAT) 2008–2009

Employment (0 = Not Employed, 1 = Employed)	M1	M2	M3	M4
Intermarried	−0.04^*^	−0.08^***^	−0.07^***^	−0.08^***^
	(0.02)	(0.02)	(0.02)	(0.02)
*Education level (Ref. low secondary)*				
Upper secondary		0.12^***^	0.12^***^	0.11^***^
		(0.02)	(0.02)	(0.02)
University		0.17^***^	0.16^***^	0.15^***^
		(0.03)	(0.03)	(0.03)
Age		0.05^***^	0.05^***^	0.05^***^
		(0.01)	(0.01)	(0.01)
Age2/1000		−0.65^***^	−0.66^***^	−0.66^***^
		(0.09)	(0.08)	(0.09)
North			0.03*	0.04**
			(0.02)	(0.02)
Dependent children			−0.11^***^	−0.11^***^
			(0.02)	(0.02)
*YSM categories (Ref. 0–5)*				
6–10				0.04*
				(0.02)
11–15				−0.02
				(0.03)
> 15				0.06
				(0.04)
Place of origin (Ref. Western Europe)				
Other non-EU				−0.07*
				(0.02)
Eastern Europe				0.01
				(0.02)
Constant	0.45^***^	−0.66^***^	−0.53^***^	−0.49^***^
	(0.03)	(0.14)	(0.14)	(0.15)
Observations	2544	2544	2544	2544

Standard errors are shown in parentheses. *, ** and *** denote that the coefficients are statistically significant at the 10%, 5% and 1% levels, respectively. The reference category for education is up to lower secondary education, and for years since migration categories, it is duration of migration from 0 to 5 years. The reference category of country of origin is Western European countries. The reference category for intermarried are endogamous

#### Robustness Checks

As detailed in the methodological section, we performed two robustness checks to ensure the reliability of our findings across different estimation methods and to better capture the relationship between intermarriage and employment. The first robustness check employs instrumental variables via a two-stage least squares (2SLS) to address the endogeneity of intermarriage. The second check investigates additional factors that may correlate with intermarriage and potentially influence employment outcomes. These factors include partner's income, homeownership, a synthetic proxy for attitudes toward women’s employment, from each partner.

#### Robustness Check 1: Addressing the Endogeneity of Intermarriage

To address potential endogeneity in intermarriage (either due to unobservable variables or reverse causality), we used an instrumental variable approach that accounts for the marriage market in the respective region of residence as an alternative to the linear probability model (LPM). The instrumental variable (2SLS) approach addresses endogeneity by using an external instrument to isolate the variation in the endogenous variable that is uncorrelated with the error term, providing more accurate and unbiased estimates of causal effects.

For an instrumental variable to be valid, it must satisfy two key conditions: relevance and exogeneity. Relevance requires that the instrument is correlated with the endogenous explanatory variable, intermarried, to ensure it can effectively predict variations in that variable. This is typically assessed in the first-stage regression, where a statistically significant association indicates relevance. Exogeneity implies that the instrument is uncorrelated with the error term in the explanatory equation, ensuring that it does not exert a direct effect on the outcome variable.

In our analysis, we use the percentage of foreign men in each region, as the proportion of foreign men reflects the availability of foreign men relative to the total male population in each region; therefore, a higher ratio is expected to be positively related to endogamous unions and negatively related to intermarriage.

Regarding the exogeneity condition, the percentage of foreign men is unlikely to directly affect women's employment due to significant occupational segregation by gender in Italy, especially among immigrants. Foreign men are typically employed in sectors like construction and manufacturing, while foreign women work in domestic and caregiving roles (Di Belgiojoso et al., 2015). This segregation minimizes both network effects and job competition between foreign men and women, supporting the validity of our instrument.

However, if the percentage of foreign men influences women's employment through an unobserved mechanism, the exclusion restriction could be violated. To address this concern, we rely primarily on ordinary least squares (OLS) estimates for our main results.

Similar to Table 2, in the instrumental variables analysis, we add control variables progressively across four models. The coefficient of the instrument percentage of foreign men has the expected sign, and it is statistically significant in the first stage. An increase in the number of foreign men correlates with a rise in endogamous marriages, attributed to the greater pool of foreign potential partners, and a decrease in intermarriages due to the relative reduction in the share of native men in the marriage market. Moreover, the F-statistic indicates that the instrument is strong enough (33 in the model with full set of controls). The relevance condition of the instrument is confirmed.

Compared with the LPMs, the coefficients of intermarriage are negative, statistically significant and larger in all models (nearly 45 percentage points of penalty in employment). These results might confirm the negative relationship between intermarriage and the probability of employment evidenced in the LPM estimations relative to endogamous women, and corroborate the hypothesis H1. The results are presented in Table 3.

**Table 3:** Robustness check 1. Instrumental variable (2SLS) estimates of employment among immigrant women in a partnership in Italy. Source: Istituto Nazionale di Statistica. Condizioni di Vita delle Famiglie con Stranieri (ISTAT) 2008–2009

Employment (0 = Not Employed, 1 = Employed)		M1	1 stage	M2	1 stage	M3	1 stage	M4	1 stage
Intermarried		−0.48**		−0.39**		−0.42*		−0.45*	
		(0.16)		(0.14)		(0.18)		(0.21)	
Pct. foreign men			−1.57^***^		−1.68^***^		−1.84^***^		−1.62^***^
			(0.21)		(0.20)		(0.29)		(0.29)
*Education level (Ref. low secondary)*									
Upper secondary				0.18***	0.20^***^	0.18***	0.20^***^	0.16***	0.16^***^
				(0.03)	(0.02)	(0.04)	(0.02)	(0.04)	(0.02)
University				0.26***	0.29^***^	0.26***	0.29^***^	0.23***	0.21^***^
				(0.05)	(0.03)	(0.06)	(0.03)	(0.05)	(0.03)
Age				0.06***	0	0.05***	0	0.05***	0.01
				(0.01)	(0.01)	(0.01)	(0.01)	(0.01)	(0.01)
Age2/1000				−0.65***	−0.02	−0.67***	−0.02	−0.68***	−0.05
				(0.10)	(0.09)	(0.10)	(0.09)	(0.10)	(0.09)
Residing in north						0	0.02	0.01	0.02
						(0.03)	(0.02)	(0.03)	(0.02)
Dependent children				−0.11***	0.01	−0.11***	0.01		
						(0.02)	(0.01)	(0.02)	(0.01)
*YSM categories (Ref. 0–5)*									
6–10								0.04	−0.01
								(0.02)	(0.02)
11–15								−0.03	−0.02
								(0.03)	(0.03)
> 15								0.06	−0.01
								(0.04)	(0.03)
*Place of origin (Ref. Western Europe)*									
Other non-EU								−0.21*	−0.38^***^
								(0.09)	(0.03)
Eastern Europe								−0.13	−0.32^***^
								(0.08)	(0.03)
Constant		1.01***	1.42^***^	−0.33	1.18^***^	−0.17	1.18^***^	0.03	1.50^***^
		(0.20)	(0.02)	(0.21)	(0.13)	(0.25)	(0.13)	(0.34)	(0.14)
Observations		2544	2544	2544	2544	2544	2544	2544	2544
*F-statistic*		59		72		40		33	

Standard errors are shown in parentheses. *, ** and *** denote that the coefficients are statistically significant at the 10%, 5% and 1% levels, respectively. The reference category for education is up to lower secondary education, and for years since migration categories, it is duration of migration from 0 to 5 years. The reference category of country of origin is Western European countries. The reference category for intermarried are endogamous

#### Robustness Check 2: Examining Additional Factors

When including husband characteristics as well as a synthetic proxy for attitude toward women’s employment controls, we can see that there is a negative and significant coefficient for intermarriage compared with endogamous women in each of the models. The coefficients for the husband’s earnings and attitudes toward women’s employment are also negative and significant in each of the models, even after including instrument for intermarriage. When including the proxy for the wife’s attitudes toward women’s employment, its coefficient appears as less significant than those of the husband, and they seem to have no significant effect at all once we take into account the endogeneity of intermarriage. This further confirms hypothesis H1 and indicates that the results of the main analysis remain robust, even when considering additional characteristics, such as husband’s earnings, attitudes toward women employment or even homeownership. The results are reported in Table 4.

**Table 4:** Robustness check 2: additional factors and instrumental variable (2SLS) analysis of employment among immigrant women in a partnership in Italy. Source: Istituto Nazionale di Statistica. Condizioni di Vita delle Famiglie con Stranieri (ISTAT) 2008–2009

Employment (0 = Not Employed, 1 = Employed)	LPM				2SLS	First Stage
Intermarried	−0.06*	−0.08**	−0.08**	−0.07*	−0.84***	
	(0.03)	(0.03)	(0.03)	(0.03)	(0.29)	
*Education level (Ref. low secondary)*						
Upper secondary	0.12***	0.11***	0.11***	0.11***	0.16***	0.07***
	(0.03)	(0.03)	(0.03)	(0.03)	(0.04)	(0.02)
University	0.20***	0.18***	0.19***	0.19***	0.29***	0.14***
	(0.04)	(0.04)	(0.04)	(0.04)	(0.06)	(0.04)
Age	0.05***	0.05***	0.05***	0.05***	0.07***	0.02**
	(0.01)	(0.01)	(0.01)	(0.01)	(0.02)	(0.01)
Age2/1000	−0.64***	−0.66***	−0.65***	−0.66***	−0.88***	−0.30**
	(0.15)	(0.15)	(0.15)	(0.15)	(0.20)	(0.13)
Residing in north	0.08***	0.08***	0.08***	0.09***	−0.01	−0.02
	(0.03)	(0.03)	(0.03)	(0.03)	(0.05)	(0.03)
Dependent children	−0.12***	−0.11***	−0.11***	−0.11***	−0.10***	0.02
	(0.02)	(0.02)	(0.02)	(0.02)	(0.02)	(0.02)
*YSM categories (Ref. 0–5)*						
6–10	0.04	0.04	0.04	0.04	0.04	0.01
	(0.03)	(0.03)	(0.03)	(0.03)	(0.04)	(0.02)
11–15	0.08*	0.08*	0.09*	0.09**	0.10*	0.02
	(0.05)	(0.05)	(0.05)	(0.05)	(0.05)	(0.04)
> 15	0.13**	0.14**	0.14**	0.14**	0.19***	0.07
	(0.06)	(0.06)	(0.06)	(0.06)	(0.07)	(0.05)
*Place of origin (Ref. Western Europe)*						
Other non-EU	−0.21***	−0.2***	−0.04	−0.04	−0.35**	−0.37***
	(0.05)	(0.05)	(0.08)	(0.08)	(0.15)	(0.07)
Eastern Europe	−0.01	0.00	0.08	0.08	−0.14	−0.26***
	(0.05)	(0.05)	(0.06)	(0.06)	(0.11)	(0.05)
Husband earnings	−0.19***	−0.20***	−0.20***	−0.2***	−0.05	0.22***
	(0.04)	(0.04)	(0.04)	(0.04)	(0.07)	(0.03)
Husband attitudes		0.12***	0.13***	0.13***	0.26***	0.16***
		(0.03)	(0.03)	(0.03)	(0.06)	(0.03)
Homeownership				−0.06*	0.07	0.17***
				(0.05)	(0.05)	(0.07)
Wife attitudes			0.11**	0.12**	0.04	−0.09**
			(0.05)	(0.05)	(0.07)	(0.04)
Pct. foreign men						−1.92***
						(0.05)
Constant	1.40***	1.29***	0.94**	0.85*	0.22	−1.04***
	(0.40)	(0.40)	(0.43)	(0.44)	(0.58)	(0.37)
Observations	1311	1311	1311	1311	1311	1311
*F-statistic*					24.10	

Standard errors are shown in parentheses. *, ** and *** denote that the coefficients are statistically significant at the 10%, 5% and 1% levels, respectively. The reference category for education is up to lower secondary education, and for years since migration categories, it is duration of migration from 0 to 5 years. The reference category of country of origin is Western European countries. The reference category for intermarried and for endogamous are those who are single

Descriptive statistics for the analyzed women are presented in Appendix Table 8. Women partnered with zero-earnings individuals were excluded.

#### Heterogeneity Analysis

To explore the mechanisms behind the negative association between intermarriage and employment, we conducted additional analyses incorporating interaction terms between marriage type (intermarriage or endogamy) and several factors including: place of origin (EU, Eastern Europe and non-EU), regional dummies accounting for unemployment rate (by region: Northeast, Northwest, Center, South and Islands), wife’s education (upper secondary or higher vs. lower education), age gap within the marriage (greater than 6 years), migration reason (family, work or other) and the presence of dependent children (age 6 or younger). To make the interpretation of the interaction terms easier, we calculate marginal effects. The results are detailed in Table 5. (The base model including interaction terms is presented in Appendix, Table 12).

**Table 5:** Marginal effects of heterogeneity in employment: LPM estimates for immigrant women in a partnership in Italy. Source: Istituto Nazionale di Statistica. Condizioni di Vita delle Famiglie con Stranieri (ISTAT) 2008–2009. https://www.istat.it/it/archivio/52405

0.intermarried (base outcome)	dy/dx	SE	t	P >| t |	[95% CI]	
1.intermarried						
*Origin*						
EU	−0.04	0.07	−0.55	0.58	−0.16	0.09
Other non-EU	−0.11	0.04	−2.98	0.00	−0.18	−0.04
Eastern Europe	−0.08	0.03	−2.53	0.01	−0.14	−0.02
*Regional unemployment*						
Nord east	0.05	0.05	1.16	0.25	−0.04	0.15
Nord west	−0.08	0.05	−1.57	0.12	−0.17	0.02
Center	−0.02	0.05	−0.44	0.66	−0.12	0.08
South	−0.11	0.05	−2.29	0.02	−0.20	−0.02
Islands	−0.25	0.05	−5.45	0.00	−0.34	−0.16
*Higher education*						
Lower education	−0.07	0.02	−2.90	0.00	−0.11	−0.02
Higher education	−0.05	0.06	−0.90	0.37	−0.16	0.06
*Age gap* > *6*						
Not higher than 6	−0.06	0.03	−1.80	0.07	−0.12	0.00
Higher than 6 years	−0.04	0.03	−1.16	0.25	−0.10	0.03
*Migration reason*						
Family	0.03	0.03	1.05	0.29	−0.03	0.09
Work	−0.23	0.03	−7.26	0.00	−0.29	−0.16
Other	−0.19	0.10	−1.99	0.05	−0.38	0.00
*Dependent children*						
Yes	−0.06	0.03	−2.10	0.04	−0.12	0.00
No	−0.12	0.03	−3.53	0.00	−0.18	−0.05

The reference category is endogamous interacting to the indicated variable

While the association between intermarriage and employment is negative among immigrant women from all origins, the magnitude of the effect varies across groups. The effect of intermarriage is not significant for women from other European countries but is particularly pronounced for those from non-EU countries (−11 percentage points) and Eastern Europe (−8 percentage points).

This disparity may be due to gender ideologies in Italy, which remain more traditional compared to most other European countries. As noted by Pessin and Arpino (2017), immigrants from traditional societies tend to adapt to progressive destinations, while those from less traditional societies may retain their views when migrating to countries with more traditional norms. In the Italian context, native partners may reinforce these traditional roles, constraining immigrant women’s labor market participation.

A process of self-selection may also be at play. Women who intermarry may choose partners with traditional ideologies, while endogamous immigrant women with progressive views may marry partners with similar preferences. Alternatively, intermarried women may marry native men with sufficient financial resources, allowing them to abstain from employment. This dynamic could further imbalance bargaining power within the couple, reinforcing traditional gender norms.

Although productivity theory and social theory suggest that intermarriage facilitates access to native networks and resources, these benefits may be undermined by the persistence of traditional gender roles (Pessin & Arpino, 2017; Scoppa & Stranges, 2019). According to bargaining power theory (Naldini & Solera, 2018), native husbands with conservative views may limit their wives’ participation in the workforce.

Therefore, rather than facilitating integration, intermarriage may hinder employment by perpetuating traditional gender ideologies and power imbalances, constraining immigrant women’s integration into the Italian labor market.

The following rows of Table 5 examine how intermarriage interacts with regional unemployment rates in Italy during the 2008 financial crisis. A notable finding is that intermarried immigrant women living in the in the South and Islands experience the largest employment penalties, at 11 and 25 percentage points, respectively, and are statistically significant. These two regions exhibited the highest unemployment rates of all the analyzed areas, suggesting that the effect of intermarriage on employment may depend on local labor market conditions and the market capacity to absorb intermarried immigrant women’s labor supply as they might compete for similar jobs with endogamously married women. However, it needs to be noted that the South and Islands are not only the regions with the highest unemployment rates, but it is also more likely that the traditional attitudes toward gender roles and female employment are more prevalent (Lomazzi, 2017). This could be another possible explanation of the difference between endogamous and intermarried migrant women in these regions.

The following rows of Table 5 show the interaction between intermarriage and women’s education. The employment penalty associated with intermarriage is slightly lower among women with higher education compared to those with lower education levels. This suggests that education is not a strong determinant of the differences in the effect of intermarriage on employment.

For the heterogeneity by age gap within couples, the data reveal that the effect of intermarriage on employment does not vary significantly depending on the age difference between partners.

Regarding the heterogeneity by migration reasons, such as family, work and other motivations, compared to endogamous women, intermarried women who migrated for work or other reasons experience notably higher employment penalties compared to their endogamous counterparts. This may suggest that, among intermarried women, the need or desire to remain employed after entering a partnership might be weaker. However, these interpretations should be treated with caution, as it is not possible to empirically determine whether the analyzed women entered a partnership/married before or after immigration, or how long after immigration partnership/marriage occurred.

Finally, the last rows of Table 5 examine the heterogeneity of the intermarriage effect on employment by the presence of dependent children. The penalty is significant for both, intermarried women with and without dependent children. However, it is larger among women without children (−12 percentage points) than among those with dependent children (−6 percentage points). This might indicate that endogamous women without children feel more the financial need or desire to be employed than intermarried women. Another possible explanation could be the presence of selection effects in having children among intermarried and endogamous couples.

### Intermarriage and the Intensity of Employment (Hours Worked)

With regard to the relationship of intermarriage with labor intensity, we did not find evidence of an association between intermarriage and labor intensity (see results presented in Table 6, Column 2). Therefore, we did not find support for our hypothesis that intermarriage is negatively related to the number of working hours (H2).

**Table 6:** Marginal effects from a hurdle model on the working hours of immigrant women in Italy. Source: Istituto Nazionale di Statistica. Condizioni di Vita delle Famiglie con Stranieri (ISTAT) 2008–2009

Hours worked	M1	M2
Intermarried	0	−0.07
	(0.37)	(0.38)
Dependent children	−0.09	−0.12
	(0.36)	(0.41)
Intermarried*Dchild (ref. Endogamos*Dchild)		−0.48
		(0.66)
Intermarried* No Dchild (ref. Endogamous *No Dchild)		0.29
		(0.43)
*Education (Ref. low secondary)*		
Upper secondary	3.39***	3.39***
	(0.78)	(0.78)
University	4.89***	4.91***
	(1.23)	(1.23)
Age	0.18***	0.18***
	(0.03)	(0.03)
Age2/1000	−2.31***	−2.32***
	(3.80)	(3.80)
*Place of origin (Ref. Western Europe)*		
Other non-EU	−1.26	−1.25
	(1.34)	(1.33)
Eastern Europe	1.26	1.29
	(1.34)	(1.34)
*YSM categories (Ref. 0–5)*		
6–10	0.51	0.5
	(0.85)	(0.85)
11–15	−1.3	−1.3
	(1.10)	(1.10)
> 15	2.34	2.32
	(1.41)	(1.41)
Residing in north	1.43*	1.42
	(0.72)	(0.72)
Observations	2544	2544

Reported coefficients represent marginal effects from a Hurdle Model

Standard errors are shown in parentheses. *, ** and *** denote that the coefficients are statistically significant at the 10%, 5% and 1% levels, respectively. The reference category for education is up to lower secondary education, and for years since migration categories, it is duration of migration from 0 to 5 years. The reference category of country of origin is Western European countries. The reference category for intermarried are endogamous. Hurdle are the marginal outcomes

#### Heterogeneity Analysis

Following the same approach as the employment extensity analysis, we conducted a heterogeneity analysis. Given the assumption that mothers of young children are more likely to work part-time, we assess whether having dependent children moderates the association between intermarriage and hours worked. This was done by including an interaction term between the marriage type and a dummy indicating the presence of dependent children (for more details please see Table 6, Column 3). As the interaction terms between intermarriage and the present of dependent children were not significant, it can be concluded that the effect of intermarriage is not moderated by the present of dependent children.

### The Effect of Intermarriage on Underemployment

We hypothesized that intermarriage has a positive effect on underemployment, because it could increase gender specialization and diminish the bargaining power of immigrant women compared with women in endogamous unions. We used LPM (Table 7, Column 2) and probit estimation (Table 7, Column 4) methods, including only the sample that reported positive hours of work. The results suggest that there is no significant relationship between intermarriage and underemployment. The hypothesis that intermarriage has a positive association with underemployment (H3) is therefore not corroborated.

**Table 7:** Underemployment among immigrant women in Italy: LPM and probit marginal effects at average. Source: Istituto Nazionale di Statistica. Condizioni di Vita delle Famiglie con Stranieri (ISTAT) 2008–2009

Underemployed (0 = Not Underemployed, 1 = Underemployed)	LPM Coefficients		Probit Marginal Effects	
Intermarried	−0.03	−0.14	−0.03	−0.15
	(0.03)	(0.78)	(0.03)	(0.26)
Dependent children	−0.09***	−0.09***	−0.09***	−0.41***
	(0.03)	(0.03)	(0.02)	(0.02)
Intermarried*No Dchild (ref. Endogamous *No Dchild)		−0.10		−0.04
		(0.06)		(0.03)
Intermarried*Dchild (ref. Endogamos*Dchild)		−0.13		−0.01
		(0.06)		(0.04)
*Education (Ref. low secondary)*				
Upper secondary	0.02	0.03	0.02	0.03
	(0.03)	(0.02)	(0.03)	(0.02)
University	−0.02	−0.02	−0.02	−0.02
	(0.04)	(0.04)	(0.03)	(0.03)
Age	0.12	0.10	0.01	0.76
	(0.10)	(0.15)	(0.01)	(1.52)
Age2/1000	−0.14	−0.02	−0.14	−0.03
	(0.14)	(0.05)	(0.14)	(0.07)
*YSM categories (Ref. 0–5)*				
6–10	0.01	0.01	0.01	0.01
	(0.03)	(0.03)	(0.03)	(0.03)
11–15	0.03	0.04	0.03	0.04
	(0.04)	(0.04)	(0.04)	(0.04)
> 15	−0.05	−0.05	−0.05	−0.05
	(0.04)	(0.04)	(0.04)	(0.04)
*Place of origin (Ref. Western EU)*				
Other non-EU	0.03	0.02	0.03	0.03
	(0.04)	(0.04)	(0.04)	(0.04)
Eastern Europe	0.09*	0.09*	0.08*	0.08*
	(0.04)	(0.04)	(0.04)	(0.04)
Constant	−0.07	0.11		
	(0.22)	(0.08)		
Observations	1055	1055	1055	1055

Standard errors are shown in parentheses. *, ** and *** denote that the coefficients are statistically significant at the 10%, 5% and 1% levels, respectively. The reference category for education is up to lower secondary education, and for years since migration categories, it is duration of migration from 0 to 5 years. The reference category of country of origin is Western European countries. The reference category for intermarried and for endogamous are those who are single

#### Heterogeneity Analysis

For a deeper understanding of the effect of intermarriage on underemployment between different groups of immigrant women, we examined the potential interaction between intermarriage and the presence of dependent children, adding the interaction term in the above regression models (see Table 7, Columns 3 and 5) The statistically insignificant interaction terms indicate that the effect of intermarriage on underemployment is not moderated by the presence of dependent children.

## Conclusions

The aim of this paper is to analyze the effect of intermarriage on employment (being employed or not), employment intensity (hours worked) and underemployment among immigrant women in Italy. The paper contributes to the scientific literature exploring the effect of intermarriage on the labor market participation of immigrant women. Analyzing employment patterns and their determinants for immigrants, particularly women, is extremely relevant due to their disadvantaged position in employment compared with natives (Ballarino & Panichella, 2017). In particular, the role of intermarriage in the integration of immigrant women into the labor market is under-researched. The current paper also contributes to the literature by exploring the mechanisms behind the association between intermarriage and the three employment outcomes. We used a specialized survey focused on the living conditions of families with foreign members in Italy.

For the main finding, the linear probability model (LPM) shows that, on average, intermarried immigrant women have an 8 percentage points lower probability of being employed compared to their endogamous counterparts. This result is reinforced by robustness checks that included partner characteristics. The negative association between intermarriage and employment persisted across various analyses, including models incorporating instrumental variables and dominant gender role attitudes.

The findings of this study differ from those of Vaalavuo and Rask (2024), who, using Finnish data, show that intermarriage yields labor market benefits, but only over a sufficiently long observation period. A local partner may facilitate economic opportunities, such as language investment, with benefits emerging in the long term. These differences may stem from variations in data sources (a single-wave cross-sectional survey versus administrative data) and observation periods (a survey snapshot versus long-term individual tracking). Additionally, country-specific factors, including contextual environments and the composition of female immigrants, likely contribute to the disparities between Italy and Finland.

On the contrary, findings of the current paper align with previous research (Ballarino & Panichella, 2018) and support productivity theory, which suggests that intermarriage leads to gender specialization, resulting in a less favorable labor market position for intermarried women. This outcome is particularly pronounced when women come from countries with traditional gender ideologies where their native husbands may reinforce a traditional division of labor. In contrast, endogamous women may adopt more progressive gender attitudes (Pessin & Arpino, 2017), facilitating their participation in the labor market. Moreover, these results align with the family investment hypothesis, which suggests that women in endogamous marriages face greater pressure to support their family’s integration. Additionally, dependency on legal status through intermarriage may further weaken the bargaining power of intermarried women, limiting their ability to engage in the labor market.

The intermarriage penalty varies significantly by education, country of origin, regional unemployment, migration reasons and the presence of dependent children. While higher educational attainment slightly mitigates the penalty, the effect is limited. The penalty is particularly pronounced for women from non-EU countries and Eastern Europe, where traditional gender norms may persist within intermarriages.

Economic conditions also play a role. The intermarriage penalty is strongest in high-unemployment regions like the South and Islands, where traditional gender roles may further reinforce the disparity. Additionally, women who migrated for work reasons face higher penalties compared to their endogamous counterparts. This suggests their work preferences may be undermined by traditional gender norms and imbalanced bargaining power within intermarriages. These women may also experience a combination of reduced financial pressure and a restrictive labor market, leaving them with few incentives to seek employment. Women who migrated due to “other” reasons present similar finding.

Interestingly, the employment penalty is larger for intermarried women without dependent children, implying that financial necessity or stronger labor market attachment drives employment more for childless endogamous women than for their intermarried counterparts. This could suggest that childless endogamous women experience a greater financial need or motivation to be employed compared to intermarried women. Another potential explanation is the possibility of selection effects influencing the decision to have children among both intermarried and endogamous couples. These interpretations warrant further empirical investigation beyond the scope of this paper.

Regarding the intensity of labor market work, we found no statistically significant differences between intermarried and endogamous women. Likewise, intermarriage did not appear to affect underemployment, suggesting that potential benefits of intermarriage may be undermined by traditional gender norms, restrictive migration laws and limited labor market opportunities.

During our analyses, we faced several limitations. First, the small sample size, especially for child migrants and second-generation immigrants, excluded them from the analysis. Second, missing data on language proficiency and marital duration limited our ability to isolate the effect of intermarriage. Third, the dataset, collected in 2008 during the post-2007 financial crisis, may reflect employment challenges unevenly across endogamous and intermarried women. Finally, the absence of country-of-origin data for many women prevented linking proxies for attitudes toward women’s employment to specific individuals.

Future research should aim to improve the precision of these estimates by analyzing a more recent and comprehensive dataset with information on language proficiency, a larger sample size and a longitudinal dimension. Identifying alternative instruments for intermarriage would also be beneficial.

To verify the external validity of our findings, similar analyses should be conducted in other Southern European countries or regions with comparable immigrant populations and different policies, allowing for meaningful comparisons.

## Appendix

See Tables 8, 9, 10, 11 and 12

**Table 8:** Descriptive statistics of immigrant women in Italy: partner characteristics by partnership type. Source: Istituto Nazionale di Statistica (ISTAT). Data from the “Condizioni di Vita delle Famiglie con Stranieri. (2008–2009)” https://www.istat.it/it/archivio/52405

Variable	Total		Endogamous		Intermarried	
	(N = 1657)		(N = 1295)		(N = 362)	
	Mean	S.D	Mean	S.D	Mean	S.D
Employment	0.41	0.49	0.41	0.49	0.41	0.49
*Education level*						
Low secondary	0.46	0.50	0.52	0.50	0.24	0.43
Upper secondary	0.43	0.50	0.40	0.49	0.53	0.50
University	0.11	0.32	0.08	0.27	0.23	0.42
Average age	35.92	8.10	35.77	8.25	36.49	7.58
Residing in north	0.50	0.50	0.52	0.50	0.41	0.49
NDC	0.49	0.66	0.49	0.67	0.50	0.63
*YSM categories*						
0–5	0.40	0.49	0.40	0.49	0.40	0.49
6–10	0.37	0.48	0.38	0.49	0.35	0.48
11–15	0.14	0.34	0.14	0.34	0.14	0.34
> 15	0.09	0.29	0.09	0.28	0.12	0.33
*Place of origin*						
Western Europe	0.08	0.27	0.03	0.18	0.24	0.43
Non-EU	0.44	0.50	0.47	0.50	0.31	0.46
Eastern Europe	0.48	0.50	0.49	0.50	0.45	0.50
Husband earnings	9.77	0.39	9.72	0.37	9.97	0.39
Husband attitudes	1.11	1.02	1.84	0.41	2.14	0.44
Homeownership	0.18	0.38	0.14	0.35	0.33	0.47
Wife attitudes	1.91	0.48	1.80	0.44	2.00	0.57
Pct. foreign men	0.09	0.04	0.10	0.04	0.09	0.04

**Table 9:** Marriage type by country of origin among immigrant women in Italy. Source: Istituto Nazionale di Statistica (ISTAT). Data from the “Condizioni di Vita delle Famiglie con Stranieri. (2008–2009)” https://www.istat.it/it/archivio/52405

Place of origin	Endogamous	Intermarried	Share of Intermarried (%)	Total
EU	80	163	67	243
Other non-EU	392	171	30	563
Albania	251	18	7	269
Poland	44	77	64	121
Rumania	422	157	27	579
Ukraine	37	47	56	84
Macedonia	43	4	9	47
Moldova	41	15	27	56
China	114	3	3	117
Philippines	56	8	13	64
India	52	1	2	53
Marocco	202	10	5	212
Tunisia	60	4	6	64
Ecuador	30	9	23	39
Peru	26	7	21	33
Total	1850	694	27	2544

**Table 10:** Balance statistics of immigrant women in Italy (ages 18–65) by migration age (child vs. adult migrants). Source: Istituto Nazionale di Statistica. Condizioni di Vita delle Famiglie con Stranieri (ISTAT) 2008–2009

Variable	Migrated as child (< 18)			Migrated as adult (18 +)			*p*-values	Test
	N = 290 (0.93%)			N = 2544 (89.77%)				
	Observations (if Var = 1)	Mean	S.D	Observations (if Var = 1)	Mean	S.D		
Intermarried	29	0.10	0.30	694	0.27	0.5	< 0.001	Fisher's exact
Employment	71	0.24	0.43	1020	0.40	0.49	< 0.001	Fisher's exact
Education level								
Low secondary	183	0.63	0.48	1153	0.45	0.50	< 0.001	Fisher's exact
Upper secondary	100	0.34	0.47	1087	0.43	0.49	0.007	Fisher's exact
University	7	0.02	0.15	304	0.12	0.32	< 0.001	Fisher's exact
Average age (median, IQR)	26 (23, 30)	27.11	5.30	36 (30, 43)	37.15	8.89	< 0.001	Wilcoxon rank-sum
Residing in north	168	0.58	0.50	1155	0.45	0.50	< 0.001	Fisher's exact
NDC (median, IQR)	1 (0, 1)	0.81	0.76	0 (0, 1)	0.47	0.66	< 0.001	Wilcoxon rank-sum
Years Since Migration (YSM)								
0–5 Years	13	0.03	0.18	951	0.37	0.48	< 0.001	Fisher's exact
6–10 Years	86	0.29	0.46	934	0.37	0.48	0.020	Fisher's exact
11–15 Years	80	0.28	0.45	376	0.15	0.35	< 0.001	Fisher's exact
> 15 Years	111	0.38	0.49	283	0.11	0.31	< 0.001	Fisher's exact
Place of origin								
Western Europe	17	0.06	0.25	243	0.10	0.29	0.041	Fisher's exact
Non-EU	158	0.54	0.50	1145	0.45	0.50	0.002	Fisher's exact
Eastern Europe	115	0.39	0.49	1156	0.45	0.50	0.062	Fisher's exact
Regional unemployment								
Northeast (3%)	91	0.31	0.46	601	0.24	0.42	0.005	Fisher's exact
Northwest (4%)	77	0.27	0.44	554	0.22	0.41	0.074	Fisher's exact
Center (6%)	54	0.19	0.39	504	0.20	0.40	0.70	Fisher's exact
South (12%)	31	0.11	0.31	450	0.18	0.38	0.002	Fisher's exact
Islands (14%)	37	0.12	0.34	435	0.17	0.38	0.067	Fisher's exact
Partner age gap > 6 years	160	0.58	0.50	856	0.34	0.47	< 0.001	Fisher's exact
Migration reason								
Family-driven migration	230	0.79	0.39	1337	0.53	0.50	< 0.001	Fisher's exact
Work-driven migration	53	0.18	0.41	1120	0.44	0.50	< 0.001	Fisher's exact
Other	7	0.02	0.15	87	0.03	0.18	0.49	Fisher's exact
Instruments								
Pct. foreign men (median, IQR)	0.12 (0.11, 0.13)	0.10	0.04	0.11 (0.03, 0.12)	0.09	0.04	< 0.001	Wilcoxon rank-sum

The column displays the median and Inter Quartile Ratio (IQR) for the variables: age, number of dependent children (NDC) and percentage of foreign men within the region (Pct. Foreign men)

P-values represent significance levels for differences between child and adult migrants. Fisher's exact test is used for categorical variables, and the Wilcoxon rank-sum test is used for continuous variables

**Table 11:** LPM estimates of employment among immigrant women in a partnership in Italy (including child migrants). Source: Istituto Nazionale di Statistica. Condizioni di Vita delle Famiglie con Stranieri (ISTAT) 2008–2009

Employment (0 = Not Employed, 1 = Employed)	M1	M2	M3	M4
Intermarried	−0.02	−0.07^**^	−0.06^**^	−0.07^***^
	(0.02)	(0.02)	(0.02)	(0.02)
*Education level (Ref. Low Secondary)*				
Upper secondary		0.11^***^	0.10^***^	0.09^***^
		(0.02)	(0.02)	(0.02)
University		0.17^***^	0.16^***^	0.15^***^
		(0.03)	(0.03)	(0.03)
Age		3.53^***^	1.04	−0.62
		(1.03)	(1.09)	(1.25)
Age2/1000		−0.10^**^	−0.10^*^	0.15
		(0.04)	(0.04)	(0.09)
North			0.04^*^	0.04^*^
			(0.02)	(0.02)
Dependent children			−0.11^***^	−0.11^***^
			(0.01)	(0.01)
*YSM categories (Ref. 0–5)*				
6–10				0.07^**^
				(0.02)
11–15				0.01
				(0.03)
> 15				0.07^*^
				(0.03)
Migrated before 18 (Ref. Migrated at Age 18 or Later)				−0.25^***^
				(0.07)
*Place of origin (Ref. Western Europe)*				
Other non-EU				−0.06
				(0.04)
Eastern Europe				0
				(0.04)
Constant	0.39^***^	0.22^***^	0.35^***^	0.41^***^
	(0.01)	(0.04)	(0.05)	(0.06)
Observations	2834	2834	2834	2834

Standard errors in parentheses. *, ** and *** denote statistical significance at the 10%, 5% and 1% levels, respectively. Reference category for education is up to lower secondary, for years since migration categories, it is 0 to 5 years. The reference category of country of origin is Western Europe. The reference category for intermarried are endogamous

**Table 12:** Heterogeneity analysis of employment among immigrant women in a partnership in Italy—main results. Source: Istituto Nazionale di Statistica. Condizioni di Vita delle Famiglie con Stranieri (ISTAT) 2008–2009

Employment (0 = Not Employed, 1 = Employed)	M1	M2	M3	M4	M5	M6
Intermarried	−0.04	0.05	−0.07**	−0.06	0.03	−0.06*
	(0.07)	(0.05)	(0.02)	(0.03)	(0.03)	(0.03)
*Origin **(Ref. Western Europe)*						
Other non-Europe	−0.03	−0.07*	−0.09*	−0.05	−0.07*	−0.07
	(0.06)	(0.04)	(0.04)	(0.04)	(0.04)	(0.04)
Eastern Europe	0.03	−0.01	−0.01	0	−0.05	0
	(0.06)	(0.04)	(0.04)	(0.04)	(0.04)	(0.04)
*Intermarried*Origin*						
Intermarried*Non-EU	−0.07					
	(0.07)					
Intermarried*Eastern-Europe	−0.04					
	(0.07)					
*Reg Unemp. **(Ref. North East)*						
North West		0				
		(0.03)				
Center		−0.01				
		(0.03)				
South		−0.05				
		(0.04)				
Islands		0.04				
		(0.04)				
*Intermarried*Reg. Unemp.*						
Intermarried*N.W.		−0.13*				
		(0.07)				
Intermarried*Center		−0.08				
		(0.07)				
Intermarried*South		−0.16*				
		(0.07)				
Intermarried*Islands		−0.30***				
		(0.06)				
Higher education			0.09*			
			(0.04)			
Intermarried*Higher Education			0.02			
			(0.06)			
Partner age gap >6				−0.13***		
				(0.03)		
Intermarried*Partner age gap>6 Years				0.02		
				(0.04)		
*Migration* *Reason*						
(Ref. Family Reasons)						
Work Reasons					0.40***	
					(0.02)	
Other Reasons					0.20**	
					(0.07)	
*Intermarried*Migration* *reason*						
Intermarried*Work					−0.26***	
					(0.04)	
Intermarried*Other					−0.22*	
					(0.10)	
Dependent child						−0.15***
						(0.02)
*Intermarried*Dependent* *child*						
Intermarried*D.C						−0.06
						(0.04)
*Education* *level* *(Ref*. *Low* *Secondary)*						
Upper secondary	0.11***	0.10***		0.10***	0.08***	0.11***
	(0.02)	(0.02)		(0.02)	(0.02)	(0.02)
University	0.16***	0.16***		0.14***	0.14***	0.16***
	(0.03)	(0.03)		(0.03)	(0.03)	(0.03)
Age	52.13***	52.24***	53.71***	46.56***	37.95***	52.84***
	(7.21)	(7.18)	(7.20)	(7.27)	(6.84)	(7.21)
Age2	−657.60***	−657.98***	−677.21***	−605.87***	−493.10***	−674.93***
	(86.59)	(86.27)	(86.40)	(86.60)	(82.08)	(86.62)
Nord	0.04*		0.05*	0.04*	0.06***	0.04*
	(0.02)		(0.02)	(0.02)	(0.02)	(0.02)
NDC	−0.11***	−0.11***	−0.11***	−0.11***	−0.11***	
	(0.02)	(0.02)	(0.02)	(0.02)	(0.01)	
*YSM* *categories* *(Ref*. *0–5)*						
6–10	0.04	0.04	0.05*	0.04*	0.06**	0.04
	(0.02)	(0.02)	(0.02)	(0.02)	(0.02)	(0.02)
11–15	−0.02	−0.02	−0.02	−0.01	0	−0.02
	(0.03)	(0.03)	(0.03)	(0.03)	(0.03)	(0.03)
> 15	0.06	0.06	0.06	0.07	0.08*	0.06
	(0.04)	(0.04)	(0.04)	(0.04)	(0.03)	(0.04)
Constant	−0.60***	−0.54***	−0.54***	−0.41**	−0.45**	−0.57***
	(0.15)	(0.15)	(0.15)	(0.15)	(0.14)	(0.15)
Observations	2544	2544	2544	2544	2544	2544

Standard errors are shown in parentheses. *, ** and *** denote that the coefficients are statistically significant at the 10%, 5% and 1% levels, respectively. The reference category for all interactions is all other excluding what is included in the interaction category. The reference category of education is up to lower secondary education, and for years since migration categories, it is duration of migration from 0 to 5 years. The reference category of country of origin is Western European countries. The reference category for intermarried are endogamous

For interaction terms: Each interaction term represents the additional effect of intermarriage within a specific group. The reference group for each interaction consists of all other categories

## Footnotes

1 For example: in the USA, Kantarevic (2004), Furtado & Song (2015), Furtado & Theodoropoulos (2009), and Furtado & Theodoropoulos (2010); in Sweden, Dribe & Lundh (2008), Dribe & Nystedt (2015), Nekby (2010), and Bevelander & Irastorza (2014); in France, Meng & Meurs (2009); in Australia, Meng & Gregory (2005); in the Netherlands, Gevrek (2009); in Germany, Nottmeyer (2010); and in Denmark, Elwert & Tegunimataka (2016).

2 Existing studies have primarily focused on a limited number of countries, such as the USA (Basu, 2015), Australia (Meng & Gregory, 2005), France (Meng & Meurs, 2009) and Sweden (Bevelander & Irastorza, 2014).

3 As this paper focuses on the intermarriage premium or penalty related to employment (being employed or not, intensity of employment measured by number of hours worked and underemployment), we will leave aside a review of the extensive empirical evidence regarding the intermarriage premium or penalty on wages (Bevelander & Irastorza, 2014; Dribe & Lundh, 2008; Dribe & Nystedt, 2015; Elwert & Tegunimataka, 2016; Furtado & Song, 2015; Gevrek, 2009; Kantarevic, 2004; Meng & Gregory, 2005; Meng & Meurs, 2009; Nekby, 2010; Nottmeyer, 2010). Instead, we focus on the literature dealing with the effects of intermarriage on being employed and the number of hours worked. This is, by contrast, relatively scarce.

4 −22%, compared with −0.3% for joint, −21% for tied, 13% for first and 16% for those who migrated alone.

5 According to the Migrant Integration Policy Index: https://www.mipex.eu/italy

6 The most numerous groups of immigrant women in 2008 were Romanians (331,066), Albanians (182,206), Moroccans (158,176), Ukrainians (120,141), Chinese (85,543), Filipinos (61,915), Moldavians (55,003), Peruvians (42,495), Ecuadorians (40,170), Serbian/Montenegrins (35,563), Tunisians (34,043), Indians (33,757), Sri Lankans (27,972), Macedonians (26,849) and Brazilians (26,814).
